# A Survey on the Perceptions of Puerto Rican Cardiologists on the Use of Smartwatches and Implantable Loop Recorders

**DOI:** 10.7759/cureus.85519

**Published:** 2025-06-07

**Authors:** Roberto J Lapetina Arroyo, Ángel S Pagán Jiménez, Diego Calo, Gabriel Horruitiner, Jose E. Lopez Ventosa, Hilton Franqui

**Affiliations:** 1 Department of Internal Medicine, Yale New Haven Hospital, New Haven, USA; 2 Department of Medicine, Cardiovascular Disease Division, University of Puerto Rico School of Medicine, San Juan, PRI; 3 Department of Cardiology, Cardiovascular Disease Division, University of Puerto Rico School of Medicine, San Juan, PRI

**Keywords:** atrial fibrillation (af), early detection of atrial fibrillation, implantable loop recorder, physician perception, smartwatches, stroke, wearable device

## Abstract

A variety of portable monitoring devices have become available and have proven to be of use in the monitoring of atrial fibrillation (AF). Implantable loop recorders (ILRs) have been shown to have great sensitivity in recording arrhythmia events in long-term arrhythmia monitoring. Recently, smartwatches have gained prominence as an unobtrusive alternative for heart rhythm monitoring. The purpose of this study was to gain a better understanding of the perception of Puerto Rican cardiologists on the use of these devices in the management of AF, particularly when compared to more established long-term devices like ILRs. We administered a survey questionnaire to cardiologists currently practicing in Puerto Rico. Demographic information was collected through participant self-reporting. Participants were defined into subgroups as follows: having a cardiac subspecialty, being associated with academia, years of practicing as a cardiologist, and cardiologist age. Commonly identified limitations to smartwatch use in cardiac monitoring were device cost (82% of respondents) and patient’s technical knowledge (81.3% of respondents). There was a tendency to prefer the use of ILRs over smartwatches in higher risk AF patients (62.5% of respondents). The main perceived benefit of these devices is their capacity to provide continuous and unobtrusive rhythm monitoring (89.6% of respondents). Attitudes towards smartwatches and ILRs were evaluated through Likert scales (1 Strongly Agree - 5 Strongly Disagree), and the non-parametric approach was chosen for their assessment. The unadjusted relationship of attitudes within these defined subgroups was evaluated through the two-sample Wilcoxon-Mann-Whitney rank-sum test. Non-subspecialized cardiologists (mean Likert Scale answer 2.19 *±* 0.83) when compared to sub-specialized cardiologists (mean Likert Scale answer 2.83 *±* 0.94) were more likely to identify device accuracy as a limitation to smartwatch use for AF detection (p = 0.0234). All other differences in Likert scale ratings evaluating device cost, reliability, and sensitivity were nonsignificant between subgroups (p > 0.05).

## Introduction

Atrial fibrillation (AF) is the most common sustained cardiac arrhythmia, affecting over 33 million people worldwide [1]. With its incidence increasing with age, it affects around 1% of patients younger than 60 years and up to 8% of those 80 years or older [2]. AF is a supraventricular tachyarrhythmia characterized by uncoordinated aberrant atrial activation leading to decline of atrial mechanical function and thus, impairing physiologic cardiac function and increasing stroke risk [2]. Data from a 2001 study showed that AF has proven to have a significant economic burden with suggested United States hospitalization costs for non-valvular AF of $1.53 billion and $235 million for pharmacotherapy [3]. A more recent study found that patients with AF had significantly higher annual healthcare spending when compared with individuals without AF [4]. Currently, with the rise in the population age and the comorbid conditions, these numbers have gotten significantly higher. For instance, in 2016 alone, AF constituted $28.4 billion of the yearly US healthcare spending [5].

According to the American Heart Association/American College of Cardiology/Heart Rhythm Society guidelines on AF management, AF can be divided into four subtypes depending on their clinical forms: paroxysmal, persistent, long-standing persistent, and permanent [6]. Paroxysmal AF terminates spontaneously or with intervention within seven days of onset. Persistent AF fails to self-terminate within seven days of onset and often requires pharmacologic or electrical cardioversion to restore sinus rhythm. Long-standing persistent AF is a persistent AF that has lasted for more than 12 months. Permanent AF refers to an AF for which a joint patient-physician decision has been made not to pursue further attempts to restore or maintain sinus rhythm [6]. AF has a wide spectrum of clinical presentations which ranges from totally asymptomatic patients to those presenting with palpitations, dyspnea, fatigue, lightheadedness, and chest pain. More serious presentations can include stroke, heart failure, or hemodynamic instability [2]. These symptoms are highly non-specific and cannot be used to diagnose AF.

The diagnostic gold standard for AF is a 12-lead electrocardiogram [2]. The 12-lead ECG or a Holter ECG monitor might be too short for AF detection. Continuous AF ECG monitoring and implantable loop recorders (ILRs) are commonly being used due to their efficiency and accuracy at detecting AF. Smartwatches have gained attention as a possible noninvasive alternative for cardiac rhythm monitoring, leading the United States Food and Drug Administration to clear the device’s accuracy in detecting AF for pre-diagnostic purposes. At present, the FDA has recommended the use of various smartwatch algorithms (e.g., Apple, Samsung, Fitbit and Withings) for screening with required confirmation by 12-lead ECG prior to medical decision-making [7]. 

The use of smartwatches in the monitoring for AF is still a debated topic and few studies have sought to determine cardiologists’ opinions regarding the subject. Given the relatively novel nature of smartwatches as heart rhythm monitoring devices, it is important to gauge professional opinions, as this can better shed light on how guideline recommendations are used in clinical practice. Moreover, the data generated by this study can potentially be helpful to the medical industry in order to further optimize the function of their devices. The purpose of this study was to create data on the perception of Puerto Rican cardiologists on the use of smartwatches in the management of AF. In doing this, we sought to better characterize how cardiologists in Puerto Rico are adapting to the evolving landscape of modern cardiology. Through our survey study, we sought to compare smartwatches with more established long-term cardiac rhythm monitoring in the form of ILRs, to further understand how smartwatches' performance in AF is perceived. The rationale behind this decision was that both ILRs and smartwatches are relatively unobtrusive and potentially long-term monitoring devices. In addition, we explored the influence of various demographic (i.e., age and sex) and professional factors (ie, practice setting, association with academia, presence of subspecialty and comfort with AF management) on Puerto Rican cardiologists’ perception of the use of smartwatches for AF monitoring.

## Materials and methods

In this cross-sectional study, a total of 48 eligible participants were recruited via email affiliation to the Puerto Rico Society of Cardiology (PRSC) and in-person attendance at the 30th annual Puerto Rico Congress of Cardiology (PRCC). Written consent and detailed instructions were included before initiating the survey questionnaire. This study was approved by the University of Puerto Rico-Medical Sciences Campus Institutional Review Board; protocol #2210058817.

Survey development 

A digital questionnaire was developed by the researchers in collaboration with the University of Puerto Rico-Medical Sciences Campus Endowment Center for Investigation on Health Services (UPR-MSC ECIHS). The survey questionnaire encompassed six fields: Sociodemographic Information, physician’s experience with Afib and monitoring devices, perception about wearable devices, physician’s recommendations of wearable devices in different clinical scenarios, perceived potential of wearable devices, and considerations regarding smartwatch recommendations. The survey consisted of 34 independent items and six follow-up questions. Of these, 12 were multiple-choice questions, 16 were based on a 5-point Agreement Likert scale, and 12 were open-ended questions meant to be answered with a single number or word. Demographic information requested included: age, sex, medical practice setting, affiliation with academia, cardiology subspecialty, and years of practice. The utilized survey questionnaire is included in the Appendices section. 

Survey distribution and administration

**Figure 1:**
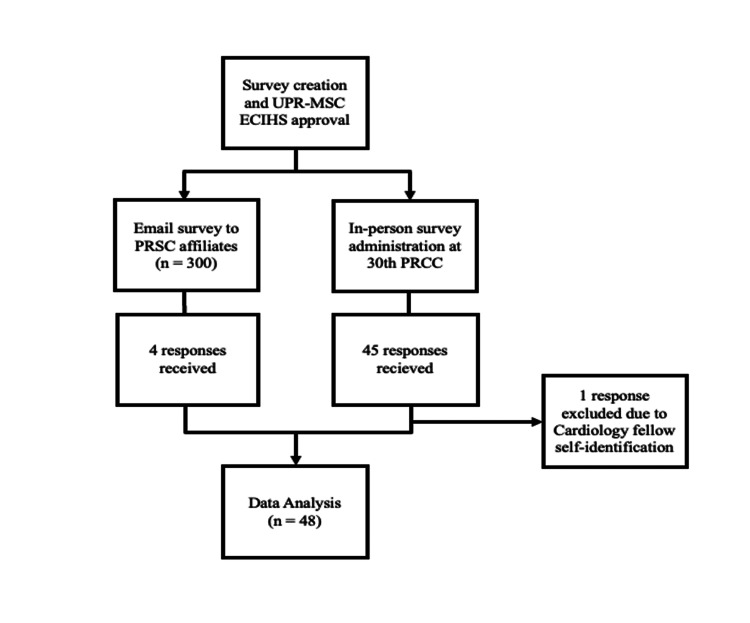
Visual depiction of the survey questionnaire step-by-step administration process. PRSC: Puerto Rico Society of Cardiologists; PRCC: Puerto Rico Cardiology Congress.

We opted for a survey that was initially sent via email to 300 addresses corresponding to cardiologists affiliated with the PRSC of which four complete responses were received. Given the inadequate number of responses received, the survey was subsequently presented in person to physicians who attended the 30th PRCC. Once the purpose of the survey study was explained to PRCC-affiliated physicians and informed consent was obtained, the agreeing participants were allowed to complete the survey independently. Participants considered for the study self-identified in the survey as cardiologists. One of the respondents self-identified as a cardiology fellow and was subsequently excluded from the study analysis. We ensured anonymity for the survey responders, and the statistician who conducted the analysis was completely blinded to any participant identifiers. Following survey distribution and removal of ineligible respondents, 48 participants were included in the statistical analysis. Figure 1 summarizes the recruitment process for this study.

Statistical analysis 

After administration of the survey, the data was statistically analyzed by the UPR-MSC ECIHS. Descriptive statistics were first used to present a profile of the participants’ demographic and professional data, their exposure to AF patients, and their perceptions of wearable devices for cardiac monitoring. All participants completed the survey tool in its totality and appropriately answered all the questions provided. Attitudes were evaluated through Likert scales, and the non-parametric approach was chosen for their assessment. Subgroup analyses were performed based on respondent groups that varied by demographic and professional characteristics and had comparable sample sizes. The unadjusted relationship of these variables with having a self-reported cardiology subspecialty was gauged through the two-sample Wilcoxon-Mann-Whitney rank-sum test. The same approach was repeated for the other relevant variables for the characterizations of cardiologists; age of 55 or older, and more than 20 years of practice. Corrections for multiple comparisons were not performed as the number of comparisons was limited and directly related to the research question. For all tests, a p-value of 0.05 or less (p-value < 0.05) was considered an indicator of a significant difference. Statistical analysis was performed using Stata v14.2 (StataCorp, Texas, USA). 

## Results

**Figure 2:**
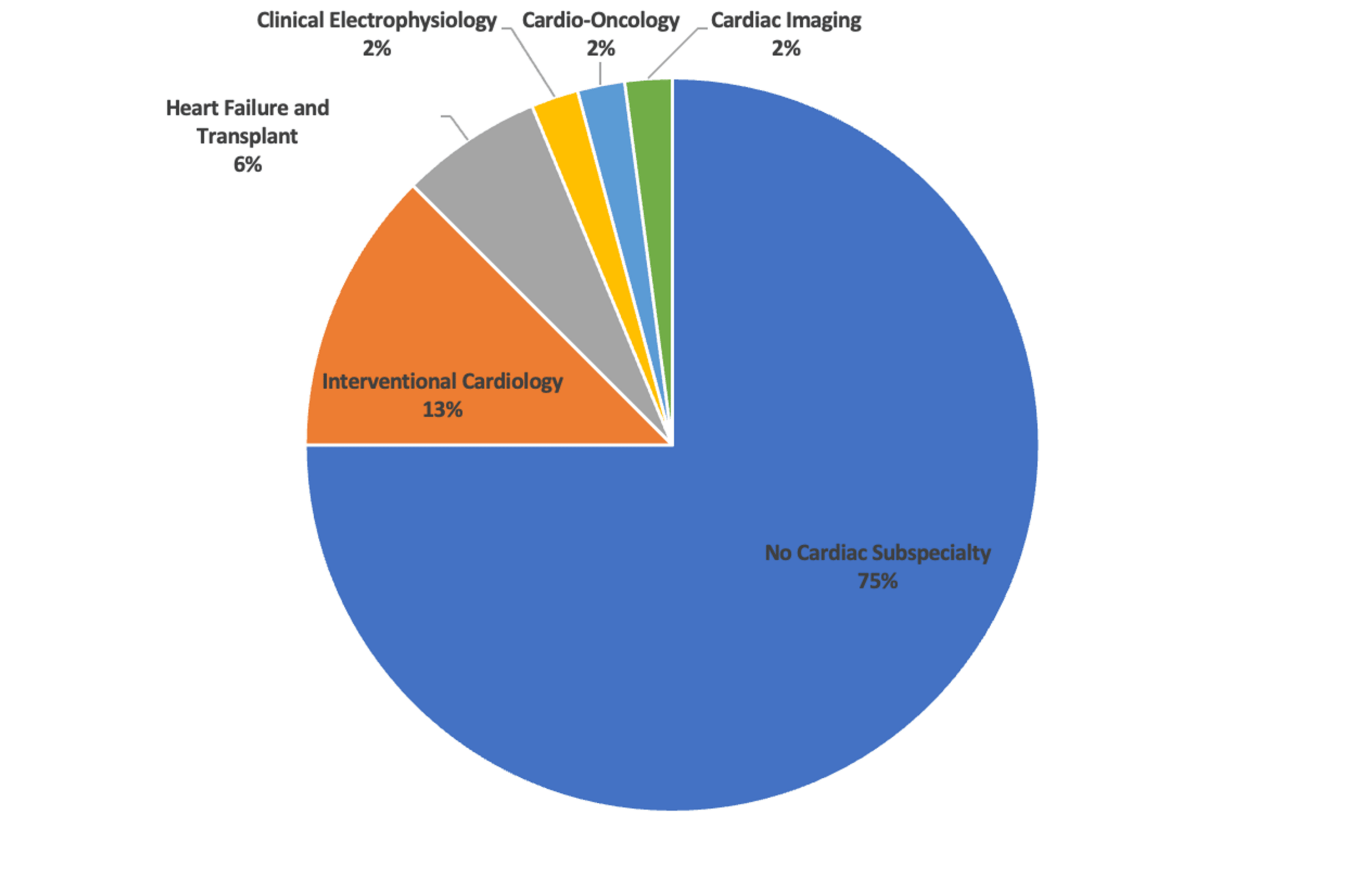
Distribution of cardiology sub-specialty based on participant self-reporting

A total of 49 Puerto Rican cardiologists responded to the survey tool. After removing a case found to be a fellowship trainee, the sample was composed of 48 participants. The demographic profile is shown in Table 1. The average age of respondents was 55.6 ± 12.4, while the ages ranged from 31 to 81. Forty-four respondents were male, while only four were female. A total of 38 respondents reported primarily working in their private office setting, while only 10 reported working primarily in a hospital setting. The average years practicing of all respondents was 21.7 ± 12.2, ranging from 1 to 44 years of practice. Nineteen respondents reported being affiliated with academia, while 29 were not affiliated with academia. A total of 12 respondents reported having an additional subspecialty, most of them being interventional cardiology (n = 6), followed by heart failure and transplant (n = 3). Figure 2 illustrates the distribution of cardiac sub-specialties based on survey-reported answers. 

**Table 1:** Demographics and Professional Profiles Based on Puerto Rican Cardiologists[' Self-Responding

Category	Variable	Value
Age	Mean ± SD	55.6 ± 12.4
	Range	31 to 81
Sex	Male (n)	44
	Female (n)	4
Medical practice setting	Hospital (n)	10
	Private Office (n)	38
Years in practice	Mean ± SD	21.7 ± 12.2
	Range	1 to 44
Academia affiliation	Yes (n)	19
	No (n)	29
Cardiologists with subspecialty	Total with Subspecialty (n)	12
	Interventional Cardiology (n)	6
	Heart Failure and Transplant (n)	3
	Clinical Electrophysiology (n)	1
	Cardio-Oncology (n)	1
	Cardiac Imaging (n)	1

The respondents’ perceptions regarding wearable devices, such as smartwatches and ILRs, are shown in Table 2. Several questions regarding wearable devices’ accuracy and reliability for detection of AF and cardiac rhythm monitoring were included in the questionnaire. Questions about possible benefits of smartwatches/ILRs in detecting paroxysmal and/or unapparent AF, convenience and unobtrusiveness were also included. The study participants successfully responded to all survey tool questions. Cardiologist attitudes were evaluated through Likert scale gradation. A high reported agreement is seen between participants on prompts referring to smartwatch accuracy in detecting AF and ILR accuracy in detecting AF, as well as both smartwatch accuracy and ILR accuracy. Notably, 81.1% of responding cardiologists agreed with the statement that smartwatches can accurately detect AF, whilst 93.7% of respondents agreed with the statement that ILRs can accurately detect AF. 87.5% of respondents agreed with the statement that ILRs are reliable alternatives for cardiac rhythm monitoring compared with 73% of respondents agreeing with the same statement when considering smartwatch use. Only 33.4% of participants agreed with the statement that smartwatches are equally as accurate as ILRs in the detection of AF. The majority of respondents to this question opted to answer Neither Agree nor Disagree (37.5%). 

**Table 2:** Cardiologist’s responses to attitude questions regarding the use of smartwatches and implantable loop recorders

	Strongly Agree	Agree	Neither Agree nor Disagree	Disagree	Strongly Disagree
Smartwatches can accurately detect Afib	n = 16 [33.3%]	N = 23 [47.8%]	N = 6 [12.5%]	N = 3 [6.3%]	N = 0 [0%]
ILRs can accurately detect Afib	N = 29 [60.4%]	N = 16 [33.3%]	N = 3 [6.3%]	N = 0 [0%]	N = 0 [0%]
Accuracy of Afib detection of a smartwatch is equal to that of ILRs	N = 1 [2.1%]	N = 15 [31.3%]	N = 18 [37.5%]	N = 14 [29.2%]	N = 0 [0%]
Smartwatches are reliable alternatives for cardiac rhythm monitoring	N = 9 [18.8%]	N = 26 [54.2%]	N = 8 [16.7%]	N = 4 [8.3%]	N = 1 [2.1%]
ILRs are reliable alternatives for cardiac rhythm monitoring	N = 16 [33.3%]	N = 26 [54.2%]	N = 2 [4.2%]	N = 3 [6.3%]	N = 1 [2.1%]
Cardiac rhythm information yielded by smartwatches should be incorporated in guideline-directed anticoagulation therapy for the management of Afib	N = 7 [14.6%]	N = 23 [47.9%]	N = 14 [29.2%]	N = 3 [6.3%]	N = 1 [2.1%]
Smartwatches are reliable for detecting paroxysmal Afib in at-risk patients	N = 10 [20.8%]	N = 26 [54.2%]	N = 9 [18.8%]	N = 2 [4.2%]	N = 1 [2.1%]
Main benefit of smartwatches is the detection of unapparent paroxysmal Afib	N = 14 [29.2%]	N = 28 [58.3%]	N = 3 [6.3%]	N = 3 [6.3%]	N = 0 [0%]
Smartwatches are an unobtrusive alternative for most patient's lives	N = 19 [39.6%]	N = 24 [50.0%]	N = 2 [4.2%]	N = 2 [4.2%]	N = 1 [2.1%]
Smartwatches are convenient because most Afib patients already use one	N = 7 [14.6%]	N = 14 [29.2%]	N = 14 [29.2%]	N = 13 [29.2%]	N = 0 [0%]

Some hypothetical scenarios were included in the questionnaire that asked respondents to choose between smartwatches, ILRs, neither of them, or if they would recommend any without a particular preference. The results are shown in Table 3. The hypothetical scenarios include preference of device in a patient with suspected paroxysmal AF (recurrent, self-resolving episodes lasting < 7 days) and in a patient with suspected persistent AF (continuous for > 7 days). Other hypothetical scenarios included preference of device for rhythm monitoring in a patient with an appropriate anticoagulation regimen with low-risk AF (defined as CHAD2DS2-VASc < 1), moderate-risk AF (CHAD2DS2-VASc = 1), high-risk AF (CHAD2DS2-VASc > 2), and in patients with a previous history of stroke. A greater proportion of Puerto Rican cardiologists would recommend monitoring with a smartwatch (48.0%) over an ILR (39.6%) for patients with paroxysmal AF. This trend was again repeated in hypothetical cases of persistent AF (52.1% favored smartwatches vs 29.2% favored ILRs). Smartwatches were also favored over ILRs in cases of low and moderate thromboembolic risk AF. ILRs were favored, however, in patients with suspected AF and a prior history of stroke (62.5% favored ILRs and 28.3% favored smartwatches). Cardiologists' preferences were more equally distributed when considering rhythm monitoring strategies in patients at high-risk of cardioembolic phenomena (33.3 % favored ILRs vs 29.2% favored smartwatches vs 27.1% favored neither). The findings reported might suggest a tendency to prefer ILRs in higher risk AF clinical scenarios (i.e., patients with higher CHAD2DS2-VASc scores or personal history of stroke). 

**Table 3:** Respondent answers to attitude questions regarding their perceptions on the use of smartwatches versus implantable loop records for cardiac rhythm monitoring

	Implantable Loop Recorder [ILR]	Smartwatch	Neither	Would recommend any without preference
Which wearable rhythm monitoring device would you prefer to use in a patient with suspected paroxysmal Afib [recurrent, self-resolving episodes lasting < 7 days]?	N = 19 [39.6%]	N = 23 [48.0%]	N = 6 [12.5%]	N =0 [0%]
Which wearable rhythm monitoring device would you prefer to use in a patient with suspected persistent Afib [continuous for > 7 days]?	N = 14 [29.2%]	N = 25 [52.1%]	N = 9 [18.8%]	N = 0 [0%]
Which wearable rhythm monitoring device would you prefer in low-risk atrial ﬁbrillation patients [CHAD2DS2-VASc < 1] on appropriate anticoagulation regimens?	N = 1 [2.1%]	N = 28 [58.3%]	N = 13 [27.1%]	N = 6 [12.5%]
Which wearable rhythm monitoring device would you prefer in moderate risk atrial ﬁbrillation patients [CHAD2DS2-VASc = 1] on appropriate anticoagulation regimens?	N = 11 [23.0%]	N = 21 [43.8%]	N = 12 [25.0%]	N = 4 [8.3%]
Which wearable rhythm monitoring device would you prefer in high-risk atrial ﬁbrillation patients [CHAD2DS2-VASc > 2] on appropriate anticoagulation regimens?	N = 16 [33.3%]	N = 14 [29.2%]	N = 13 [27.1%]	N = 5 [10.4%]
Which wearable rhythm monitoring device would you prefer for cardiac rhythm monitoring in patients with a history of stroke?	N = 30 [62.5%]	N = 10 [28.3%]	N = 6 [12.5%]	N = 2 [4.2%]

Table 4 shows results about possible limiting factors that respondents might consider for the recommendation of smartwatches for monitoring AF. Limiting factors asked in the questionnaire included accuracy and sensitivity of recorded data, cost of device and patient’s age, technological knowledge, and adherence to use of device. The answers were evaluated through Likert scales. Notably, 56.3% of responding cardiologists identified the accuracy of smartwatches as a limiting factor to their use, with 31.3% favoring Neither Agree nor Disagree for this statement. Patient's technical knowledge and device cost seemed to be the most commonly perceived limiting factors for smartwatch use, with 81.2% and 81.3% of participants agreeing respectively. However, a greater proportion of participants favored Strongly Agree when considering patient's technical knowledge as a risk factor to smartwatch use (43.8%). Patient age and the sensitivity of recorded data were less commonly identified as limiting factors to smartwatch use (62.5% and 52.1%, respectively). 

**Table 4:** Respondent answers to attitude questions identifying possible limiting factors for the recommendation of smartwatches for monitoring atrial fibrillation

	Strongly Agree	Agree	Neither Agree nor Disagree	Disagree	Strongly Disagree
Accuracy of recorded data is a limiting factor	N = 7 [14.6%]	N = 20 [41.7%]	N = 15 [31.3%]	N = 5 [10.4%]	N = 1 [2.1%]
Cost of the device is a limiting factor	N = 20 [41.2%]	N = 19 [40.0%]	N = 8 [16.7%]	N = 1 [2.1%]	N = 0 [0%]
Sensitivity of recorded data is a limiting factor	N = 7 [14.6%]	N = 18 [37.5%]	N = 14 [29.2%]	N = 8 [16.7%]	N = 1 [2.1%]
Patient’s age is a limiting factor	N = 14 [29.2%]	N = 16 [33.3%]	N = 13 [27.1%]	N = 4 [8.3%]	N = 1 [2.1%]
Patient’s technological knowledge is a limiting factor	N = 21 [43.8%]	N = 18 [37.5%]	N = 5 [10.4%]	N = 4 [8.3%]	N = 0 [0%]
Patient’s adherence to usage is a limiting factor	N = 12 [25.0%]	N = 23 [47.9%]	N = 7 [14.6%]	N = 6 [12.5%]	N = 0 [0%]

The two-sample Wilcoxon-Mann-Whitney rank-sum test p-value results for the four subgroup analyses are shown in Table 5. Values were assigned to each Likert scale gradation such that Strongly Agreed = 1, Agreed = 2, Neither Agree or Disagree = 3, Disagree = 4 and Strongly Disagree = 5. A subgroup median value was calculated from all the obtained responses and submitted to the non-parametric two-sample rank sum test in order to determine if there was a statistically significant difference between median values (p50). The only statistically significant difference between medians observed was between subspecialized and non-subspecialized cardiologists when considering if the accuracy of recorded data was a limiting factor to smartwatch use (p-value = 0.0323). The median value for the Likert scale answers for non-subspecialized cardiologists was 2 and 3 for specialized cardiologists, such that non-subspecialized cardiologists agreed with the statement that accuracy is a limiting factor to smartwatch use for cardiac monitoring more frequently. No other statistically significant differences were reported between subgroup means (p-value > 0.05).

**Table 5:** Two-sample Wilcoxon-Mann-Whitney rank-sum test p-value subgroup analysis results based on attitude question responses

Attitude Item	Subspecialized vs. Nonsubspecialized	Academia Affiliation vs. No Affiliation	≤20 vs. >20 Years of Practice	<55 vs. ≥55 Years of Age
Identified as Limiting Factors				
Accuracy of recorded data	0.0323	0.9378	0.4519	0.895
Cost of the device	0.5797	0.2561	0.4709	0.4806
Sensitivity of recorded data	0.5147	0.4023	0.5119	0.2407
Patient’s age	0.9001	0.9474	0.6864	0.8283
Patient’s technological knowledge	0.8138	0.7079	0.7102	0.7540
Patient’s adherence to usage	0.1334	0.7949	0.7451	0.6883
Identified as Potential Benefits				
Smartwatches are reliable in detecting paroxysmal Afib in at-risk patients	0.5799	0.6179	0.4272	0.4168
Main benefit is detection of unapparent paroxysmal Afib	0.8621	0.4511	0.4336	0.2383
Smartwatches are unobtrusive in most patients’ lives	0.9613	0.9534	0.6595	0.9174
Smartwatches are convenient since most Afib patients already use one	0.7420	0.5918	0.1012	0.3424

## Discussion

The Puerto Rican Society of Cardiology estimates its membership to be close to 325 cardiologists [8], of which close to 15% (N=48) are represented in this survey study. There is limited data on the demographic characteristics of Puerto Rican cardiologists. However, a recent study on the demographic characteristics of cardiologists in the United States evidenced a male preponderance of cardiologists [9], like what was observed in this study. The sample obtained in this survey study serves to attest to a relative underrepresentation of female cardiologists in Puerto Rico.

 Moreover, the mean age of academic cardiologists in the continental United States has been estimated at 53.5 years for male cardiologists and 48.3 years for female cardiologists [10]. The mean age of the cardiologists who responded to this study was slightly older (55.6 ± 12.4). This discrepancy could reflect the existence of an older physician workforce in Puerto Rico. Although not its primary endpoint, this study has served to begin defining the baseline demographic characteristics of Puerto Rican cardiologists through participant self-reporting. Further studies are needed to better categorize the demographic characteristics of Puerto Rican cardiologists.

The general attitude of Puerto Rican cardiologists towards smartwatch use in AF monitoring was found to be positive. 81% of respondents attested that smartwatches can accurately detect AF. 73% of respondents agreed that smartwatches are reliable alternatives for most patients. Prior survey studies have suggested that physicians from various specialties have shown a considerable degree of confidence in smartwatches, as irregular pulse notifications often led to further diagnostic testing and interventions [11]. Of the 31 cardiologists surveyed in the aforementioned study, 41.9% had recommended smartwatch use to patients [11]. A survey study evaluating patient attitudes toward smartwatches in cardiac monitoring found that 68.9% of surveyed patients expressed an interest in smartwatch cardiac monitoring [12]. Additionally, 90% of interviewed patients said they would seek medical attention based on aberrant device readings [12].

The main benefit of smartwatch monitoring as perceived by Puerto Rican cardiologists appeared to be related to their continuous and relatively comfortable rhythm monitoring capacities. 89.6% of respondents agreed with the statement that there are unobtrusive rhythm monitoring alternatives for most patients. Moreover, 87.5% of responding cardiologists agreed with the statement that the main benefit of smartwatches lies in their capacity to reliably detect AF in at-risk patients. Although the impact AF burden has on thromboembolic events remains a contested topic, paroxysmal AF has been shown to increase the yearly risk of stroke and systemic embolization events. The Atrial Fibrillation Clopidogrel Trial with Irbesartan for Prevention of Vascular Events (ACTIVE W) has suggested a yearly stroke risk of 2% for patients with paroxysmal AF [13] while the Stroke Prevention in Atrial Fibrillation Study (SPAF) has suggested yearly stroke risks of 3.2% for patients with intermittent AF [14]. Thus, the detection of paroxysmal AF remains an important issue in stroke prevention and highlights the potential of smartwatch use in AF management.

When considering limitations of smartwatch use, the most frequently perceived factor by Puerto Rican cardiologists was device expense, with 82% of responding cardiologists identifying cost as a limiting factor to use. Smartwatches for cardiac monitoring are not currently covered by any major healthcare plan in Puerto Rico. Any patient interested in this form of rhythm monitoring must therefore pay out-of-pocket for the device. Evidently, this poses a barrier to healthcare access that may limit smartwatch cardiac monitoring to patients of higher socioeconomic status. 

Patients’ technical knowledge was also frequently identified as a limiting factor to smartwatch use, with 81.3% of respondents agreeing with the statement. Moreover, 63.3% of responding cardiologists agreed that age is a limiting factor to smartwatch use. This fact is likely related to a supposition that older patients could be less technologically adept than younger patients. A survey on patient attitudes toward smart technology in cardiac rhythm monitoring found that 71% of its respondents identified the complexity of the technology as a limiting factor to its use [12]. Complexity was identified as a barrier to use particularly amongst older patients [12]. Conversely, younger patients (< 65 years) with underlying AF were more likely to be interested in smart device monitoring than older patients [12].

Although the majority of responding cardiologists responded favorably to the use of smartwatches in the detection of AF, 53% of respondents identified accuracy as a limiting factor to smartwatch use. Moreover, Wilcoxon-Mann-Whitney testing of subgroups only showed statistically significant differences between median values of the subgroup comparing subspecialized and non-subspecialized cardiologists (p = 0.0323; 3 and 2 respectively). Note that non-subspecialized cardiologists were found to identify the accuracy of smartwatches as a limiting factor more frequently than subspecialized cardiologists. This finding could be explained by exposure to novel technologies through additional training following a general cardiology fellowship. This difference could also be explained by increased professional exposure to smartwatch devices given the nature of electrophysiology practice or increased referrals from other medical specialties for arrhythmia detection or monitoring. Additionally, differential answering of this question could reflect differences in practice patterns. For example, one study found that electrophysiologists were more likely to employ long-term electrocardiogram monitoring when compared to non-electrophysiologist cardiologists [15]. Past survey studies have also found that patients may be apprehensive of smartwatch accuracy, with only 52% of patients trusting smartwatch accuracy [12]. Despite these findings, recent studies [16,17] have identified smartwatches as reliable alternatives with relatively high accuracy. A recent meta-analysis of 18 heterogeneous studies has found a pooled Positive Predictive Value and Negative Predictive Value of 85% and 100% respectively [18]. This could suggest that Puerto Rican cardiologists may underestimate the accuracy of smartwatches in arrhythmia detection and monitoring due to relative device novelty. Efforts through clinical training and continued medical education may be warranted to raise awareness on the usefulness of smartwatches for AF management. 

When comparing smartwatches to ILRs, Puerto Rican cardiologists appeared to favor smartwatch use more strongly in lower risk patients (i.e., patients with lower CHA2DS2-VASc scores) than in higher risk patients (i.e., patients with higher CHA2DS2-VASc scores and history of strokes). This finding is likely explained by a belief that higher risk patients merit more invasive rhythm monitoring modalities. Such presumed attitudes may be reflective of recent professional guidelines where ILRs are recommended for secondary thromboembolic prevention in patients with inconclusive non-invasive monitoring [19]. However, smartwatch performance has been tested against more established methods of cardiac monitoring. One study compared the performance of the Garmin smartwatch to simultaneous Holter monitoring and reported a sensitivity of 97.3% and specificity of 88.6% [20]. These high sensitivity values could help establish smartwatches as reliable screening tools for the monitoring of AF. In patients with persistent AF, smartwatches have also been shown to accurately assess heart rates compared to Holter rates when < 110 bpm [21]. Note however, that there is limited data comparing the relative accuracies of smartwatches with ILRs for AF monitoring. Future prospective cohort studies can be aimed at determining how these two specific modalities compare in AF detection.

The performed survey study has various limitations. Firstly, of the estimated 300 members of the Puerto Rican Society of Cardiology, only 48 (15%) answered the survey study. A larger sample size would have allowed for a more representative sample with which to make more precise assumptions about Puerto Rican cardiologist's perception. Low response rate and possible response bias limit the generalizability of this study. Moreover, given the self-enrolling nature that is innate to survey studies, the cardiologists who chose to answer the survey could have stronger opinions toward smartwatches than those who chose not to answer the survey. The information analyzed was based on physician self-reporting and estimation. Additionally, four responses were obtained via email and 44 were obtained via voluntary in-person administration of the survey. There could have been differential responses based on the setting in which the survey was administered. These two population subsets were not individually analyzed. However, all attendees of the PRSC Conference are assumed to be members and may still constitute the originally intended study population. Moreover, we opted for a convenience sampling method which could have introduced bias into the methodology of our study. In-person completion of the survey may have also introduced bias from the Hawthorne effects, although participants were allowed to independently complete the survey and answers were collected anonymously. Statistical analyses were not adjusted for baseline demographic characteristics, but multivariable analysis allowed for gauging how these demographic factors impacted participants' answers. The administered survey was not pre-tested but was validated by the UPR-MSC ECIHS. Finally, only one of the cardiologists who responded to the survey study self-identified as a Clinical Electrophysiologist. Greater representation of this subspecialty would have been ideal given their expertise in cardiac rhythm monitoring. 

## Conclusions

This survey study has helped begin to define the baseline demographic characteristics of cardiologists in Puerto Rico. There may be a notable underrepresentation of the female sex amongst cardiologists in Puerto Rico. Given high agreement rates on Likert Scale questionnaires, there is a tendency towards favorable attitudes regarding the use of smartwatches in atrial fibrillation monitoring amongst Puerto Rican cardiologists. Notably, study's generalizability is limited given low response rates. The main perceived benefit of these devices is their capacity to provide continuous cardiac monitoring in a relatively unobtrusive manner. The most frequently identified limitation for smartwatch use in cardiac rhythm monitoring was the cost of the device. Patient’s technical knowledge was also frequently identified as a barrier to smartwatch use. There was a tendency to prefer ILR use over smartwatch use in high-risk patients based on Likert Scale questionnaire answers. The only significant difference in the distribution of respondent attitude questions was observed regarding the accuracy of smartwatches within the subspecialized vs non-subspecialized respondent cardiologists. Subspecialized cardiologists identified device frequency as a barrier to use less frequently than non-subspecialized cardiologists. This discrepancy may be explained by possible exposure to novel technologies through additional training. The findings of this study highlight the need for continued education of cardiology professionals regarding novel cardiac monitoring technologies. 

## Appendices

Survey tool 

Part I: Sociodemographic Information

Let's start the interview with general demographic details.

1. What is your age? Please use a single number. _____

2. What is your biological sex?

a. Male

b. Female

c. Prefer not to answer

3. How would you define your medical practice?

a. Private office setting

b. Hospital setting

4. Are you currently affiliated with academia? (i.e., teaching position, researcher)

a. Yes

b. No

5. Do you currently practice a cardiology subspecialty?

a. Yes (please specify) ___________

b. No

6. To this day, how many years have you been practicing as a cardiologist? Please estimate a single number. _______

Part II. Physician’s Experience With Atrial Fibrillation and Monitoring Devices

Please answer the following questions based on what you've encountered in your current medical practice.

7. On average, how many patients per month with atrial fibrillation do you see? Please estimate a single number. _______

8. Of these patients with atrial fibrillation, what percentage initially arrived at your office or at the emergency department due to receiving an irregular rhythm notification via their smartwatch? Please estimate a single number. _______

9. On average, how many patients per month do you see because of emergency department referrals due to newly-onset atrial fibrillation? Please estimate a single number. _______

10. In total, to approximately how many patients with atrial fibrillation do you currently provide care? Please estimate a single number. _______

11. Of these, to what percentage did you suggest monitoring their cardiac rhythm with a smartwatch? Please estimate a single number. _______

12. Of these, to what percentage did you suggest monitoring their cardiac rhythm with an ILR? Please estimate a single number. _______

13. In the past year, how many patients have you referred for implantation of an ILR? Please estimate a single number. _______

Part III. Perception About Wearable Devices

The following statements and questions will gauge your impressions of wearable health devices. Choose the answer that best describes your opinion. For this, remember there are no right or wrong answer.

14. Smartwatches can accurately detect atrial fibrillation in most patients.

a. Strongly agree

b. Agree

c. Neither agree nor disagree

d. Disagree

e. Strongly disagree
 

15. Implantable loop recorders (ILRs) can accurately detect atrial fibrillation in most patients.

a. Strongly agree

b. Agree

c. Neither agree nor disagree

d. Disagree

e. Strongly disagree

16. The accuracy of atrial fibrillation detection of a smartwatch is equal to that of an implantable loop recorder (ILR).

a. Strongly agree

b. Agree

c. Neither agree nor disagree

d. Disagree

e. Strongly disagree

17. Smartwatches are reliable alternatives for cardiac rhythm monitoring in most patients.

a. Strongly agree

b. Agree

c. Neither agree nor disagree

d. Disagree

e. Strongly disagree

18. Implantable loop recorders (ILRs) are reliable alternatives for cardiac rhythm monitoring in most patients.

a. Strongly agree

b. Agree

c. Neither agree nor disagree

d. Disagree

e. Strongly disagree

Part IV. Recommendations of Wearable Devices by Clinical Scenario

19. Which wearable rhythm monitoring device would you prefer to use in a patient with suspected paroxysmal atrial fibrillation (recurrent, self-resolving episodes lasting < 7 days)?

a. Implantable loop recorder

b. Smartwatch

c. Neither

20. Which wearable rhythm monitoring device would you prefer to use in a patient with suspected persistent atrial fibrillation (continuous for >7 days)?

a. Implantable loop recorder

b. Smartwatch 

c. Neither

21. Which of these smartwatches (if any) would you feel most comfortable recommending to a patient with atrial fibrillation for cardiac rhythm monitoring?

a. Apple Smartwatch

b. Samsung Galaxy Watch

c. Fitbit

d. Other (Please specify) ____________

e. Would recommend any without preference

f. Would not recommend any

22. Which of these ILRs (if any) would you feel most comfortable recommending to a patient with atrial fibrillation for cardiac rhythm monitoring?

a. Medtronik ILR

b. Abbott ILR

c. Biotronik ILR

d. Other (Please specify) ______

e. Would recommend any without preference

f. Would not recommend any

23. Which wearable rhythm monitoring device would you prefer in low-risk atrial fibrillation patients (CHAD2DS2-VASc < 1) on appropriate anticoagulation regimens?

a. Implantable loop recorder

b. Smartwatch

c. Would recommend any without preference

d. Would not recommend any

24. Which wearable rhythm monitoring device would you prefer in moderate risk atrial fibrillation patients (CHAD2DS2-VASc = 1) on appropriate anticoagulation regimens?

a. Implantable loop recorder

b. Smartwatch

c. Would recommend any without preference

d. Would not recommend any

25. Which wearable rhythm monitoring device would you prefer in high-risk atrial fibrillation patients (CHAD2DS2-VASc > 2) on appropriate anticoagulation regimens?

a. Implantable loop recorder

b. Smartwatch

c. Would recommend any without preference

d. Would not recommend any

26. Which wearable rhythm monitoring device would you prefer for cardiac rhythm monitoring in patients with a history of stroke?

a. Implantable loop recorder

b. Smartwatch

c. Would recommend any without preference

d. Would not recommend any

27. Cardiac rhythm information yielded by smartwatches should be incorporated in guideline-directed anticoagulation therapy for the management of patients with atrial fibrillation.

a. Strongly agree

b. Agree

c. Neither agree nor disagree

d. Disagree

e. Strongly disagree

Part V. Perceived Potential of Wearable Devices

Choose for each statement the best fit for your level of agreement with it.

28. Smartwatches are reliable for detecting paroxysmal atrial fibrillation in at-risk atrial fibrillation patients.

a. Strongly agree

b. Agree

c. Neither agree nor disagree

d. Disagree

e. Strongly disagree
 

29. For me, the main benefit of smartwatches in the monitoring of atrial fibrillation is the detection of otherwise clinically inapparent paroxysmal atrial fibrillation.

a. Strongly agree

b. Agree

c. Neither agree nor disagree

d. Disagree

e. Strongly disagree

30. Smartwatches are an unobtrusive alternative for most patient's daily lives.

a. Strongly agree

b. Agree

c. Neither agree nor disagree

d. Disagree

e. Strongly disagree

31. Recommending a smartwatch for cardiac monitoring is convenient given that a significant amount of my atrial fibrillation patients already use a smartwatch.

a. Strongly agree

b. Agree

c. Neither agree nor disagree

d. Disagree

e. Strongly disagree

Part VI. Considerations Regarding Smartwatch Recommendations

This is the last part. Here, choose for each item the option that best fits your level of agreement:

Consideration

Likert Scale Gradation

33. Cost of the device

○

Strongly agree

○

Agree

○

Neither agree nor disagree

○

Disagree

○

Strongly disagree

34. Patient's age

○

Strongly agree

○

Agree

○

Neither agree nor disagree

○

Disagree

○

Strongly disagree

35. Patient's technological knowledge

○

Strongly agree

○

Agree

○

Neither agree nor disagree

○

Disagree

○

Strongly disagree

36. Patient's adherence and hours of usage

○

Strongly agree

○

Agree

○

Neither agree nor disagree

○

Disagree

○

Strongly disagree

37. The accuracy of the recorded data

○

Strongly agree

○

Agree

○

Neither agree nor disagree

○

Disagree

○

Strongly disagree

38. The device's sensitivity (capacity to detect relevant data)

○

Strongly agree

○

Agree

○

Neither agree nor disagree

○

Disagree

○

Strongly disagree
